# Manifold absolute pressure estimation using neural network with hybrid training algorithm

**DOI:** 10.1371/journal.pone.0188553

**Published:** 2017-11-30

**Authors:** Mohd Taufiq Muslim, Hazlina Selamat, Ahmad Jais Alimin, Mohamad Fadzli Haniff

**Affiliations:** 1 Apt Touch Sdn. Bhd., Taman Universiti, Skudai, Johor, Malaysia; 2 Centre for Artificial Intelligence & Robotics (CAIRO), Universiti Teknologi Malaysia, Kuala Lumpur, Malaysia; 3 Mechanical & Manufacturing Engineering Faculty, Universiti Tun Hussein Onn Malaysia, Parit Raja, Batu Pahat, Johor, Malaysia; Southwest University, CHINA

## Abstract

In a modern small gasoline engine fuel injection system, the load of the engine is estimated based on the measurement of the manifold absolute pressure (MAP) sensor, which took place in the intake manifold. This paper present a more economical approach on estimating the MAP by using only the measurements of the throttle position and engine speed, resulting in lower implementation cost. The estimation was done via two-stage multilayer feed-forward neural network by combining Levenberg-Marquardt (LM) algorithm, Bayesian Regularization (BR) algorithm and Particle Swarm Optimization (PSO) algorithm. Based on the results found in 20 runs, the second variant of the hybrid algorithm yields a better network performance than the first variant of hybrid algorithm, LM, LM with BR and PSO by estimating the MAP closely to the simulated MAP values. By using a valid experimental training data, the estimator network that trained with the second variant of the hybrid algorithm showed the best performance among other algorithms when used in an actual retrofit fuel injection system (RFIS). The performance of the estimator was also validated in steady-state and transient condition by showing a closer MAP estimation to the actual value.

## Introduction

Electronic fuel injection (EFI) system is expected to be one of the most promising technologies on improving the fuel economy and reducing harmful emissions [1]. One way to achieve this is by accurately estimating the engine load. There are several types of fuel injection methods being used in modern system of a spark ignition (SI) engine. The most commonly used are the air-flow method or speed-density method. Both methods require estimation of the engine load by estimating the air mass flow rate (AMF) into the engine cylinder [2]. In the air-flow method, the estimation of cylinder AMF are based on the measurement of the mass air flow (MAF) sensor near the throttle plate. But, in speed-density method, the estimation of the cylinder AMF were based on the measurement by a manifold absolute pressure (MAP) sensor by using combination of look-up tables or polynomial expressions [3].

There are several efforts made by past researchers in estimating the absolute pressure of the engine’s intake manifold. The analytical approach as in [4–6] focus on the degree by degree detail variation of the engine parameters and components, typically in mathematical equations that represent the physical characteristics of that engine. This lead to model that represent some components of the engine such as the manifold itself. Other popular approaches such as the Mean Value Engine Model (MVEM) in [7] and Kalman filter in [8] also lead to estimation of manifold pressure. However, the detail physical equations that describe the analytical model can often become fairly complicated, which makes it difficult to apply in real-engine application. With the advancement of computing technology, empirical approach such as artificial neural network was adapted in estimating the manifold pressure as described in [9]. The neural network approach generally uses the experimental data to predict most of the engine process.

In this paper, a different approach in estimating the manifold absolute pressure of a small engine were introduced by using a feedforward neural network with hybrid training algorithm. This approach only uses two inputs (throttle position and engine speed) that does not require additional sensor or the MAP sensor to estimate the absolute pressure. The Neural network were chosen because of its capability of learning underlying input/output relationship without requiring the development of an explicit model of the underlying relationship [10, 11]. Furthermore, the training algorithm consists of several algorithms which are Levenberg-Marquardt (LM), Bayesian Regularization (BR) and Particle Swarm Optimization (PSO)) that merged together to compensate the drawbacks of each other.

The outline of this paper is as follows. Section 2 discuss on several algorithms that was use in this study to train the neural network. In section 3, the proposed feedforward network with hybrid training algorithm is presented. While in Section 4, discusses on the performance analysis of each combination of training algorithm on both simulation and experimental works. Lastly, section 5 conclude all the works presented in this paper.

## Training algorithm for a multi-layer feedforward neural network

**Fig 1:**
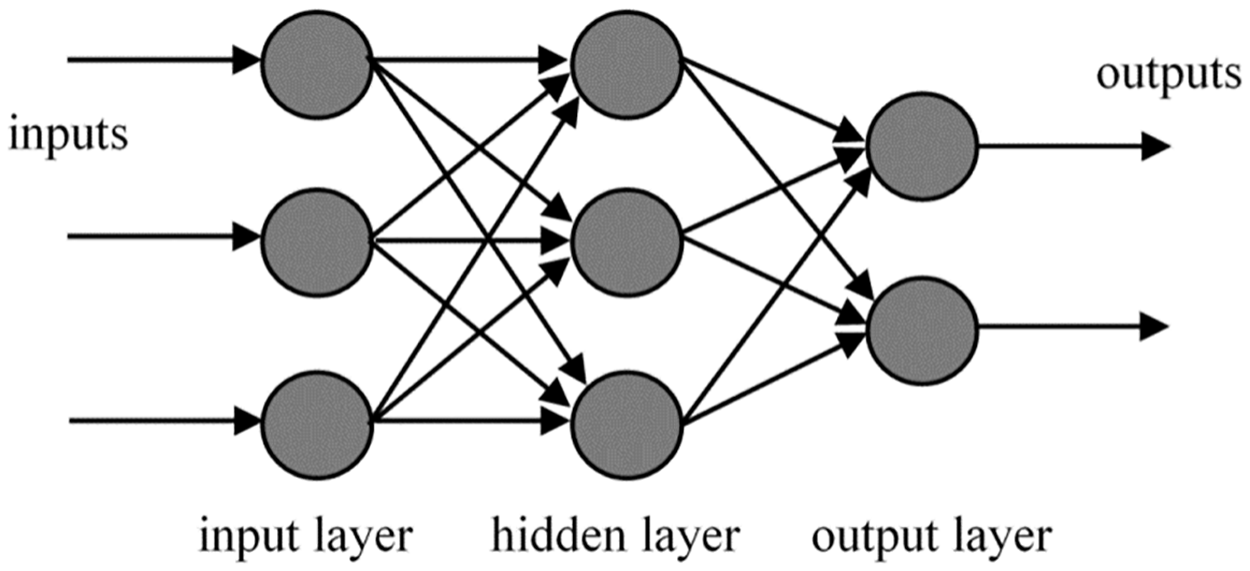
The structure of multi-layer feedforward network.

Artificial neural networks (ANN) mimics the human brain nerves and neurons. It consists of densely interconnected computer processors which works in parallel [12]. ANN in most cases can alter its internal structure based on the inside and outside information that feed through the network during the learning phase. ANN consist of an input layer, one or more hidden layer and an output layer. Fig 1 shows the general structure of an ANN. For feedforward networks, the mean squared error (*MSE*) is usually used as the performance function. *MSE* is the average squared error between the network output, *t* and the target output, *o* which is represented by (1).

$$MSE\;=\;\frac{1}{2}\sum_{i\;=\;1}^{N}{\left(t^{i}-\;o^{i}\right)}^{2}$$ (1)

In this study, 3 different algorithms were used together to train the neural network, which are Levenberg-Marquardt (LM), Bayesian Regularization (BR) and Particle Swarm Optimization (PSO). This will increase the robustness and the performance of the feedforward network which is discussed in section 3 later. Each of the algorithms are presented as follows.

### Levenberg-Marquardt (LM)

LM algorithm is often used in minimizing a nonlinear function [13,14]. The LM algorithm is a combination of the steepest descent method and the Gauss-Newton method. This means, it combines the stability of the steepest descent method and the speed advantage of the Gauss-Newton algorithm in reducing the sum of the squared error by using a different *λ* values in solving (2).

$$(J^{t}J\;+\;\lambda I)\delta \;=\;J^{t}E$$ (2)

Where *J* is the Jacobian matrix, *λ* is the damping factor and *δ* represent the weight update vector that the user must find. Next, *E* is the error vector which is produced by each of the input used in the network training. The value of *δ* exhibit on how much the user want to change the network weight in order to achieve better performance. The *J*^*t*^*J* matrix is known as the approximated Hessian, which is shown in (3).

$$H\;\approx \;J^{t}J$$ (3)

**Fig 2:**
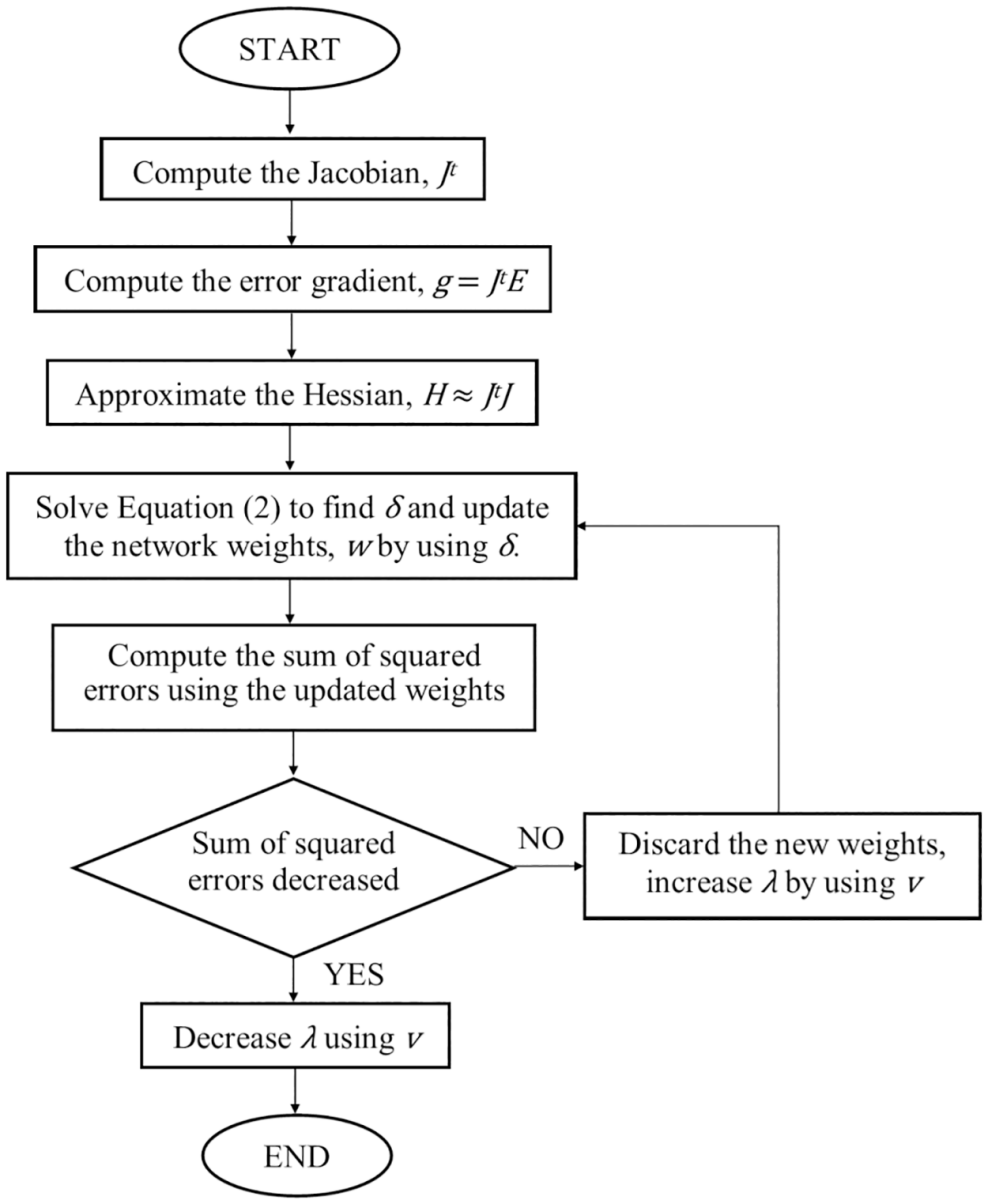
The flow chart of LM algorithm.

The value of *λ* is adjusted by using an adjustment factor, *v* which referred as 10. If *λ* needs to be decreased, it will be divided by *v*. However, if *λ* needs to be increased, it will be multiplied by *v*. The entire procedure is repeated until there is a decrease in the error which indicates the end of the current iteration. Fig 2 illustrate on the steps computed by LM algorithm in each learning iteration.

The performance of LM algorithm depends on how well the training procedure is planned. Poor planning will lead to poor network generalization and overfitting issues. There are several training procedures effectively been used with LM. One of the easiest way is by using regularization method as describe in the next section.

### Bayesian Regularization (BR)

BR algorithm can improve the network’s generalization, avoid overfitting and also eliminate the need of a costly cross validation method [15]. During learning process, BR utilize the cost function to find the minimal error by using the minimal weights. The direction for the learning process which either towards the minimal error or towards minimal weight is determined by the two Bayesian hyper-parameters, *α* and *β* which are described in (4) and (5). A third variable *γ* shown in (6), can point out the complexity of the network by showing the number of effective weights being used.

$$\alpha \;=\;\gamma \;/\;2E_{w}$$ (4)

$$\beta \;=\;(N\;-\;\gamma )\;/\;2E_{d}$$ (5)

$$\gamma \;=\;W\;–(\alpha \;*\;tr(H^{-1}))$$ (6)

Where *N* is the total number of training data. *W* is the total number of weights and biases.

*tr*(*H*^*-1*^) is the trace of the inverse Hessian matrix. This results in a cost function as follows:

$$C\;=\;\beta E_{d}\;+\;\alpha E_{w}$$ (7)

**Fig 3:**
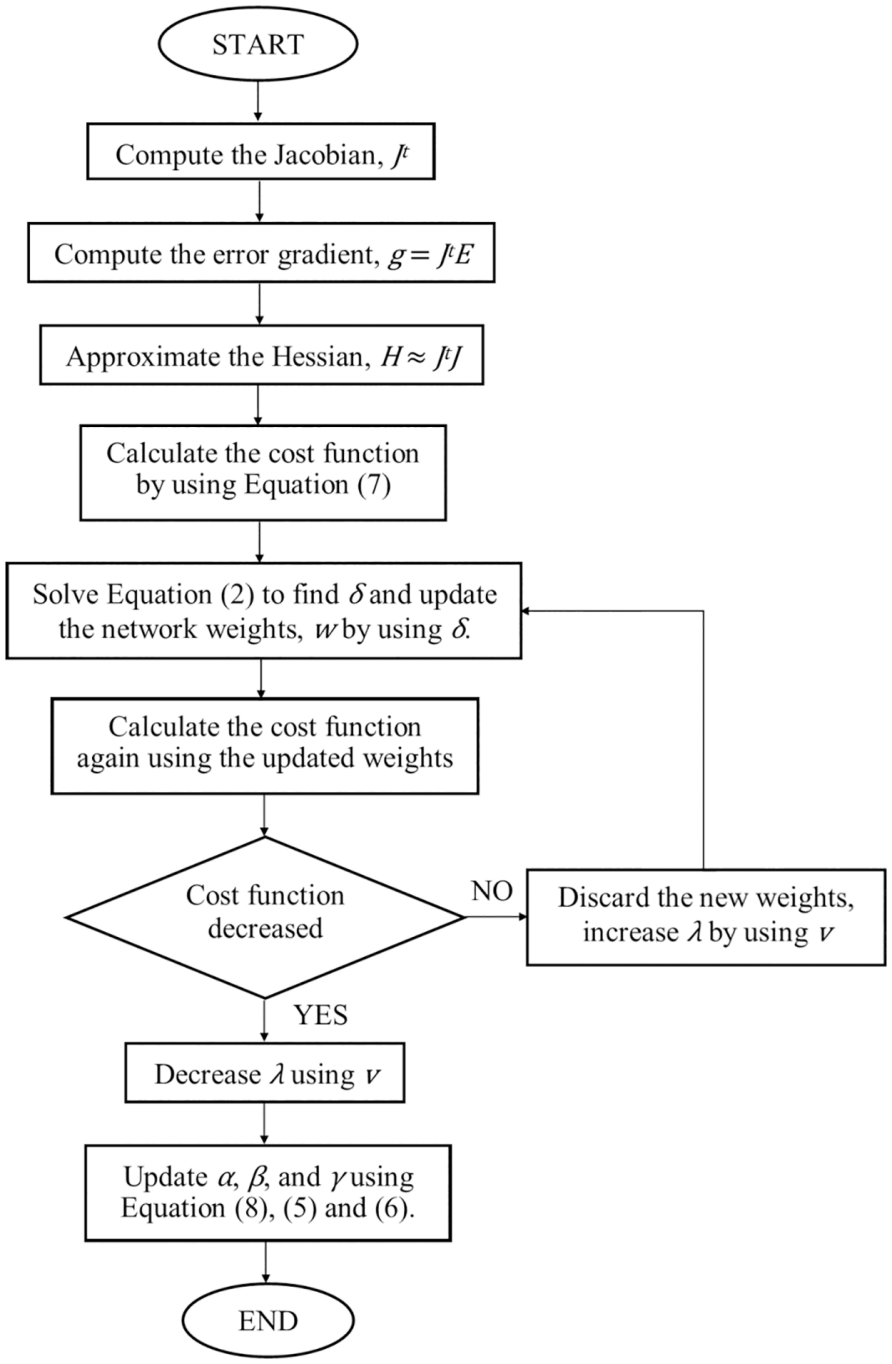
The flow chart of LM with BR algorithm.

Where *E*_*d*_ is the sum of squared errors and *E*_*w*_ is the sum of squared weights. In case of having a small training data, [16] introduced a modified Bayesian update equation of variable *α* to solve the iteration deficiency problem in the existing algorithm. Fig 3 shows the steps computed by LM with BR in each learning iteration.

$$\alpha \;=\;W\;/\;(2E_{w}\;+\;tr(H^{-1}))$$ (8)

### Particle Swarm Optimization (PSO)

The concept of Particle Swarm Optimization (PSO) algorithm has been used widely in the engineering applications because of its easy implementation which requires fewer computational memories [17]. PSO also has a fast rate of convergence and a powerful global searching ability. In PSO algorithm, a swarm of n particles within a searching space will search globally for optimal solution. The following (9) and (10) are used to update the position vector and velocity vector of the particle *i* from iteration *t* to the next iteration *t*+1.

$$x_{i,j}(t\;+\;1)\;=\;x_{i,j}(t)\;+\;v_{i,j}(t\;+\;1),\;j\;=\;(1,\ldots ,d)$$ (9)

$$v_{i,j}(t\;+\;1)\;=\;wv_{i,j}(t)\;+\;c_{1}r_{1}[p_{i,j}–x_{i,j}(t)]\;+\;c_{2}r_{2}[p_{g,j}–x_{i,j}(t)]$$ (10)

**Fig 4:**
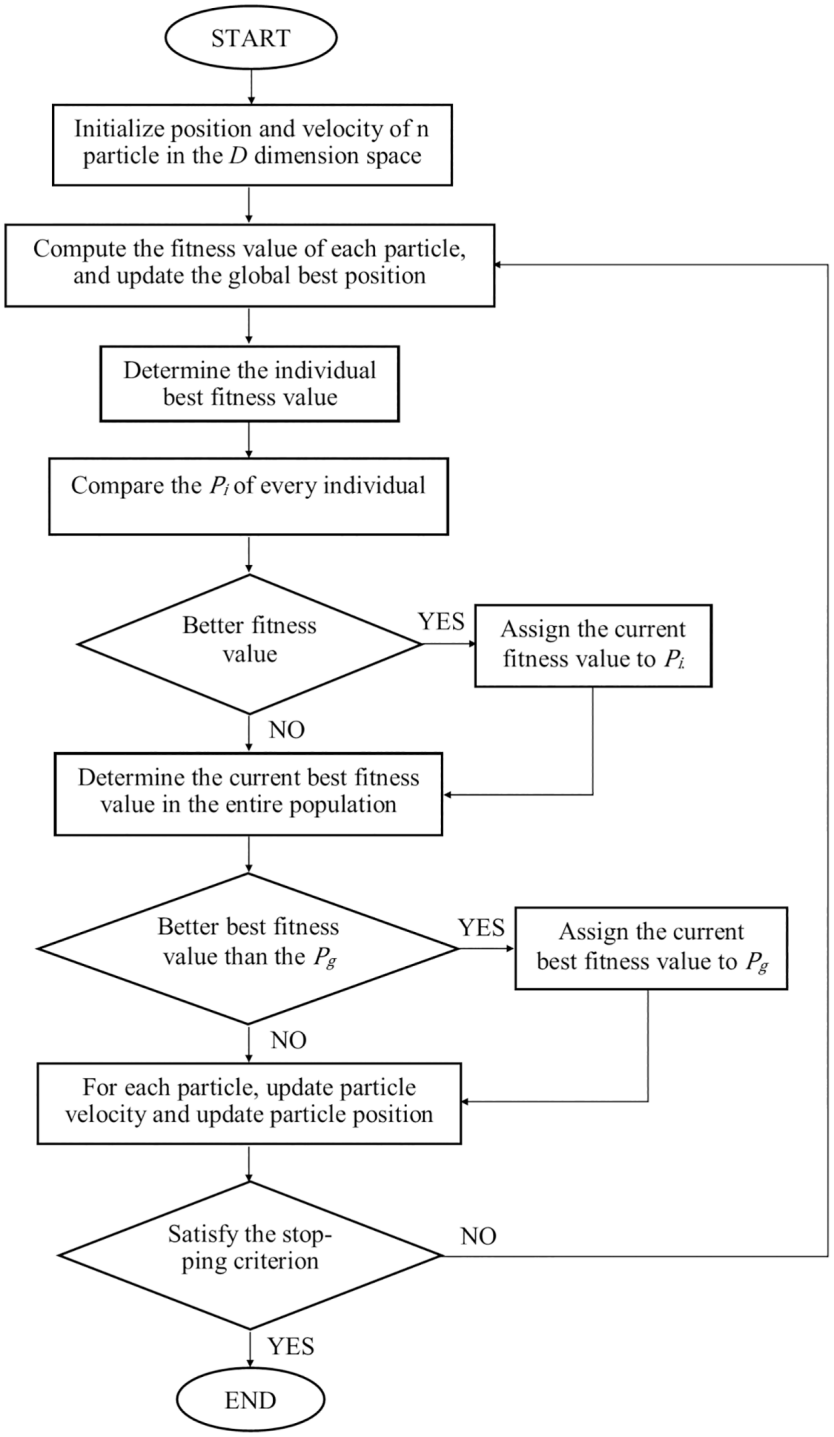
The basic flow chart of PSO algorithm.

Where *X*_*i*_ = (*x*_*i*,*1*_, *x*_*i*,*2*_, …, *x*_*i*,*j*_, …, *x*_*i*,*d*_) are the position vector, *V*_*i*_ = (*v*_*i*,*1*_, *v*_*i*,*2*_, …, *v*_*i*,*j*_, …, *v*_*i*,*d*_) are the velocity vector, *P*_*i*_ = (*p*_*i*,*1*_,*p*_*i*,*2*_, …, *p*_*i*,*j*_, …, *p*_*i*,*d*_) are the personal best position vector, *P*_*g*_ = (*p*_*g*,*1*_, *p*_*g*,*2*_, …, *p*_*g*,*j*_, …, *p*_*g*,*d*_) are the global best position vector and *w* is the varying inertia factor. *c*_*1*_ and *c*_*2*_ are the acceleration coefficients. *r*_*1*_ and *r*_*2*_ are the random number between 0 and 1. Fig 4 shows the basic flow chart of PSO algorithm.

## Manifold absolute pressure (MAP) estimation using neural network with hybrid training algorithm

**Fig 5:**
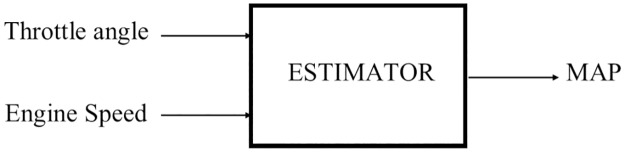
Two-stage neural network estimator block diagram.

The MAP estimator is based on a multi-layer feed forward neural network which uses the mean squared error (*MSE*) as its performance function. This network has one hidden layer with hyperbolic tangent sigmoid transfer function and a linear transfer function in the output layer. The network block diagram is illustrated in Fig 5.

Based on Fig 5, the network consists of two inputs (throttle angle and engine speed), and one output (MAP). One hidden layer network were used to avoid complication in the network structure and high computation time. The LM algorithm is extremely dependent on the initial weights of the network which caused an inconsistent convergence to local minima [18]. In order to cater the drawbacks of LM algorithm and improve on the performance of the neural network, a hybrid training algorithm were developed with the addition of the BR and the PSO algorithms. Regularization is one of the methods that was used in the neural network to improve network generalization and to avoid overfitting.

LM and BR are only good in exploring the local region for solution. To solve this problem, PSO were used so that a global search procedure can be done. Neural network can also be trained by using the standard PSO as presented in section 4. Even though in some cases, PSO tends to converge prematurely before reaching an optimum solution [19] but together with LM algorithm and BR algorithm this drawback can be avoided.

In this study, there are two methods of PSO that is being used with LM and BR. The results of both methods are presented in section 4. The term PSO_a_ and PSO_b_ will be used to characterize the first and the second method. In the first method, the weight initialization is optimized before the network training by using PSO. This method is called the hybrid algorithms (LM+BR+PSO_a_). It is expected that, by optimizing the weight initialization before the training, using the PSO, the network converge much faster and has better performance. This is due to the better start point of the initial network weights and eventually reduces any wasted computation by the training algorithm in search for new weights for better network performance.

Below are the steps computed by the hybrid algorithm (LM+BR+PSO_a_) in neural network training:

1. Randomly initialize position (network weights) and velocity of n particles (networks).
2. By using *MSE* as the fitness function, optimize the network weights (particles) with PSO.
3. Train the network by using LM with BR using the optimized weights according to the flow chart described in Fig 3.

In the second method, the PSO were used to initialize the network weights and validating *MSE* in each iteration. This method is called the hybrid algorithm (LM+BR+PSO_b_). At the end of each iteration, the *MSE* is validated before changing or updating the current network weights for the next iteration. By doing this, the networks weight will gradually optimize from lower to higher iteration and the chance of the hybrid algorithm to search for the next best local optima is possible in each iteration. Through this method, the computation time will certainly increase, and will lead to a better network performance.

Below are steps computed by the hybrid algorithm (LM+BR+PSO_b_) in neural network training:

1. Randomly initialize position (network weights) and velocity of n particles (networks).
2. By using weights obtained from PSO, train the network by using LM with BR according to the flow chart described in Fig 3.
3. Compute *MSE* (fitness function for PSO) using trained weights from step 2.
4. If *MSE* has decreased, keep the updated weights and update the particles’ position and velocity by using (9) and (10).
5. Else, discard the updated weights and update the particles’ position and velocity using (9) and (10).
6. Repeat Step 2–5 until a number of iterations is satisfied.

### MAP estimation using simulated engine model

In this simulation work, the data sets are obtained from simulation model given in MATLAB 2013a [20] as summarized in Table 1. The main objective of the simulation is to investigate the effectiveness of the hybrid algorithm. The offline training were implement in batch mode, which the weights are updated after all the inputs in the training sets are applied to the network. There are two phases in the development of the neural network, which are training phase and testing phase. The data set are divided into two parts (837 for training phase and 93 for testing phase).

**Table 1:** Data set for neural network training.

*Data set*	*Number of patterns*	*Unit*
*Throttle angle*	*930*	*degrees*
*Speed*	*930*	*rad/s*
*Manifold Absolute pressure (MAP)*	*930*	*kpa*

### MAP estimation using a real engine

**Fig 6:**
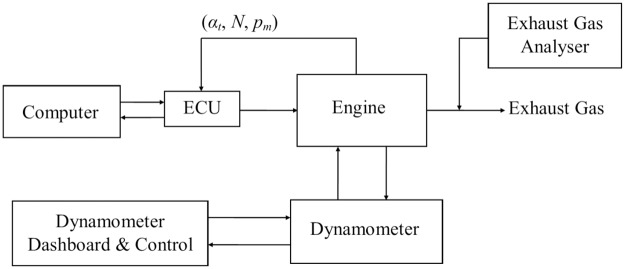
Experimental setup block diagram for data collection in RFIS.

The offline training of the estimator network were conducted by using the experimental data taken from a retrofit fuel injection system (RFIS) of a small engine as described in [21]. The main objective of this experimental work is to verify the effectiveness of the proposed estimator that are trained with the hybrid algorithm. For collecting the experimental data, a Mainline Dynolog Dynamometer system and test bench are used. A Motorcycle SYM E-BONUS 110 is used in the RFIS. The experimental setup diagram is illustrated as in Fig 6.

By referring to Fig 6, the engine is operated at different speeds and the engine input data which are the throttle angle (*α*_*t*_), engine speed (*N*) and manifold absolute pressure (*p*_*m*_) are logged into the Engine Control Unit (ECU). The ECU then logged the data into a computer for training the estimator using the proposed methods. There are 1480 data collected from the RFIS as listed in S1 Table. 1332 out of 1480 are used for offline network training. Which leaves 148 data for testing the trained network.

## Results and discussions

This section is divided into 3 subsections. Section 4.1 discussed on the selection of the neuron number for the hidden layer of network by comparing the performance of several algorithms including the hybrid algorithm with different number of neurons. Whereas in Section 4.2 discussed on the performance analysis of the MAP estimator in simulation work and Section 4.3 discussed on the performance analysis of the MAP estimator in a real engine.

### Selection of the hidden layer neuron number

A training was conducted on one of the hidden layer network of the MAP estimator with the number of neurons of 2, 4, 6 and 8. Due to the different test *MSE* produced by different sets of network weights, the networks were trained multiple of times. This series of training was known as the number of trials (*m*). Then, the average test *MSE* were recorded for detailed analysis. The testing *MSE* were used instead of the training *MSE* because the test data were never seen by the trained network. This training method was repeated and conducted on the 5 algorithms. The smallest size (number of neurons) structure that can still provide a good fitting accuracy and generalize well were selected in the end. The setting parameters of each algorithm were summarized in Table 2. The setting parameters in Table 2 were then set in such a way to ensure a fair number of network trainings for each algorithm. Optimum setting for PSO were chosen based on the study in [22,23].

**Table 2:** Setting for each algorithm for networks training.

*Algorithm*	*Setting parameters*
*LM*	*m = 50*
*LM+BR*	*m = 50*
*PSO*	*n = 5; t = 10; c_1_ = 1.49618; c_2_ = 1.49618; w = w(1–1/t)*
*LM+BR+PSO_a_*	*n = 5; t = 10; c_1_ = 1.49618; c_2_ = 1.49618; w = w(1–1/t)*
*LM+BR+PSO_b_*	*n = 5; t = 10; c_1_ = 1.49618; c_2_ = 1.49618; w = w(1–1/t)*

**Fig 7:**
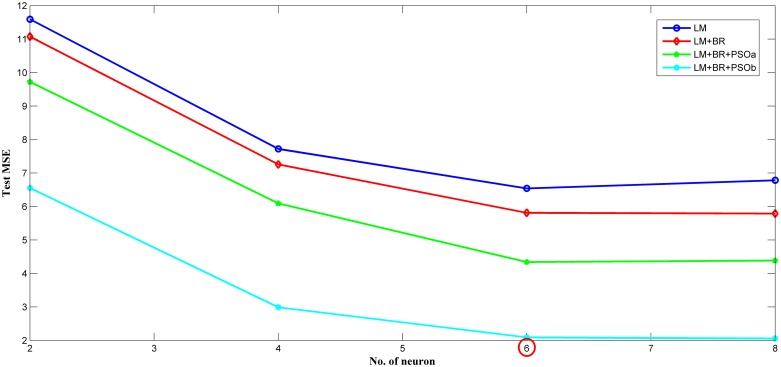
Variation of the network (MAP estimator) test MSE with the number of hidden neuron for four training algorithms.

Based on Table 2, *m* is the number of trials, *n* is the number of PSO particles, *t* is the number of iteration (number of weight restarts), *c*_*1*_ and *c*_*2*_ are the acceleration coefficients and *w* is the varying inertia factor that begins with the value of 1.4 and end with the value of 0. As the test *MSE* of the network trained with the standard PSO algorithm for each number of neurons were higher than the other algorithms, it was omitted from the plot in Fig 7. Thus, Fig 7 showed the variation of the network test *MSE* of the MAP estimator with the number of hidden neuron for four algorithms. According to Fig 7, the test *MSE* become smaller as the number of neurons increases. Noted that no more significant improvement was made for more neurons for all four algorithms. Thus, the suitable number of neurons for the MAP estimator was six.

### Performance analysis of the estimator in simulation

The training was conducted on the network using five training algorithms. The parameter of each algorithm were set using the same values, as stated in Table 2. The training procedures of each algorithm ran 20 times and the average values of the test *MSE* were computed, as well as the average computation time. This was done to get a stable and more accurate test *MSE* value. The results are summarized in Table 3 below.

**Table 3:** Performance of the MAP estimator in 20 runs.

*Training Algorithm*	*Average test MSE*	*Average computation time(s)*
*LM*	*6.1446*	*64.1941*
*LM+BR*	*1.7704*	*273.1597*
*PSO*	*249.4320*	*171.3177*
*LM+BR+PSO_a_*	*0.1088*	*336.6474*
*LM+BR+PSO_b_*	*0.0871*	*513.0515*

Based on the results present in Table 3, regardless of the computation time, when PSO were used as the training algorithm for the network, resulting in a highest average test *MSE* with 249.4320 which indicates the poorest performance among the networks with different sets of training algorithms. This also indicates an overfitting and poor generalization of the network. Followed by LM (6.1446) and LM+BR (1.7704) which had a much better network performance. As for the standard LM, the performance is much lower compared to LM and BR combined. From this results, the LM algorithm are likely to stay stuck in the local optima while failed to search for global optima as a better solution.

The hybrid algorithm (LM+BR+PSO_a_) with an average test *MSE* of 0.1088 were found to be better than both LM and LM+BR. This indicates that the network converge much faster due to PSO_a_ by having a better starting point of the initial network weights. Nevertheless, the superiority of the hybrid algorithm (LM+BR+PSO_b_) was proven with having the lowest average test *MSE* of 0.0871. Unlike PSO_a_, PSO_b_ optimized the initial weight and validate the *MSE* in every iteration towards better solution. Although, the hybrid algorithm (LM+BR+PSO_b_) produced the best network, the computation time needed in its training and testing phase was higher compared to others which was 513.0515s. However, this was not a crucial problem in this study as the training was done offline.

### Performance analysis of the estimator in a real engine

The proposed MAP estimator was applied in an actual small engine fuel injection system. The analysis was divided into 3 parts. First, was the offline analysis of the estimator performance after training with experimental data. In this part, the estimator network training was conducted by using 5 training algorithms. Similarly, the analysis method and the same parameters setting were used for all algorithms as described in simulation work (Table 2). In addition, the coefficient of determination, *R*^*2*^ between outputs and actual outputs was also computed to demonstrate the degree of prediction or fit, in the data. Second part was the online analysis of the proposed estimator output in a steady-state condition. Finally, the third part was the online analysis of the proposed estimator output in transient condition. Here, the actual absolute pressure measured by the MAP sensor was later compared with the MAP estimator output.

**Fig 8:**
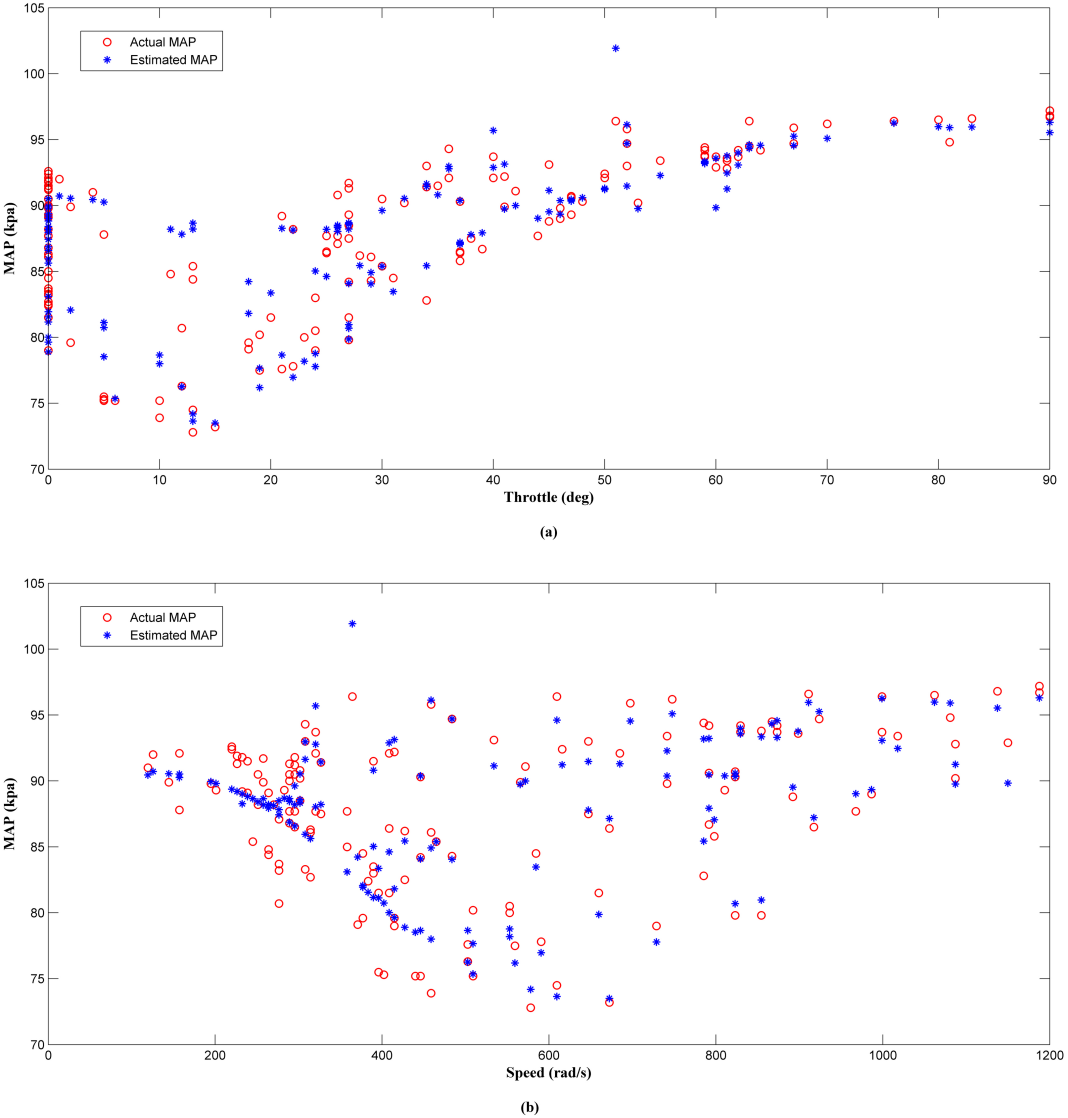
Comparison of the MAP estimator output (LM) and actual MAP as a function of (a) throttle and (b) speed.

**Fig 9:**
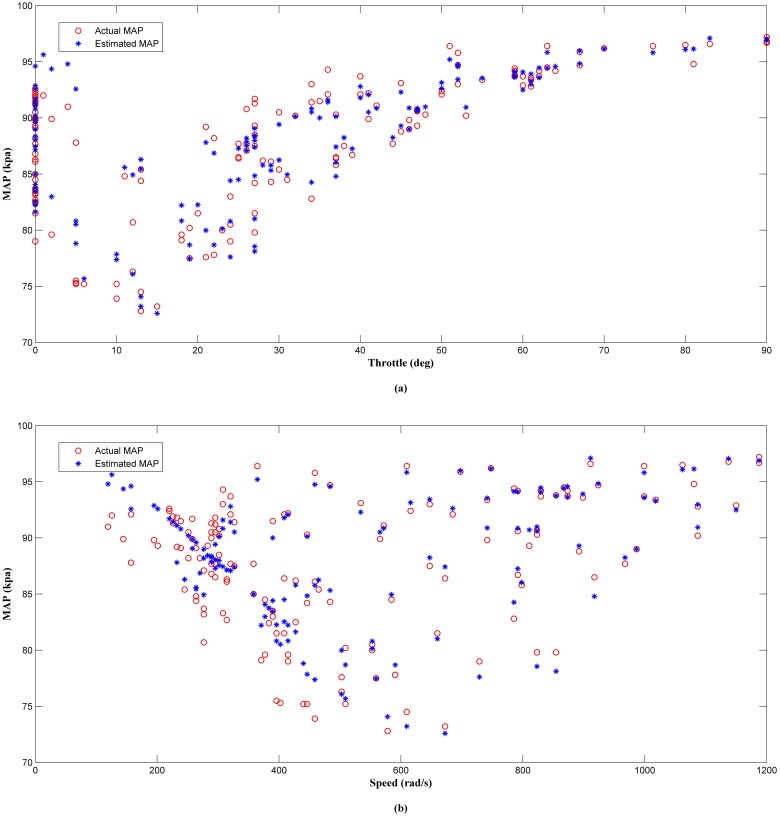
Comparison of the MAP estimator output (LM+BR) and actual MAP as a function of (a) throttle and (b) speed.

**Fig 10:**
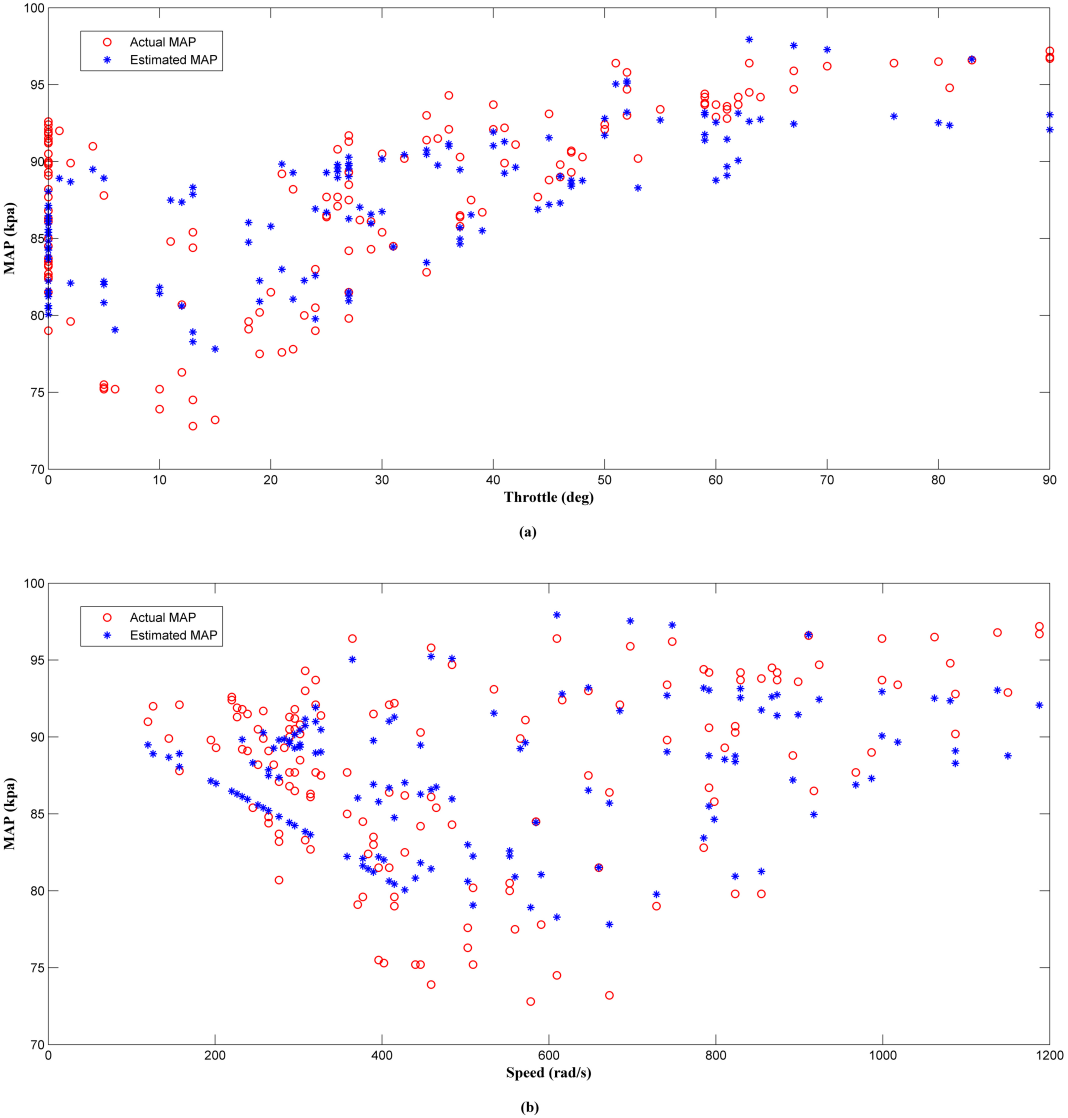
Comparison of the MAP estimator output (PSO) and actual MAP as a function of (a) throttle and (b) speed.

**Fig 11:**
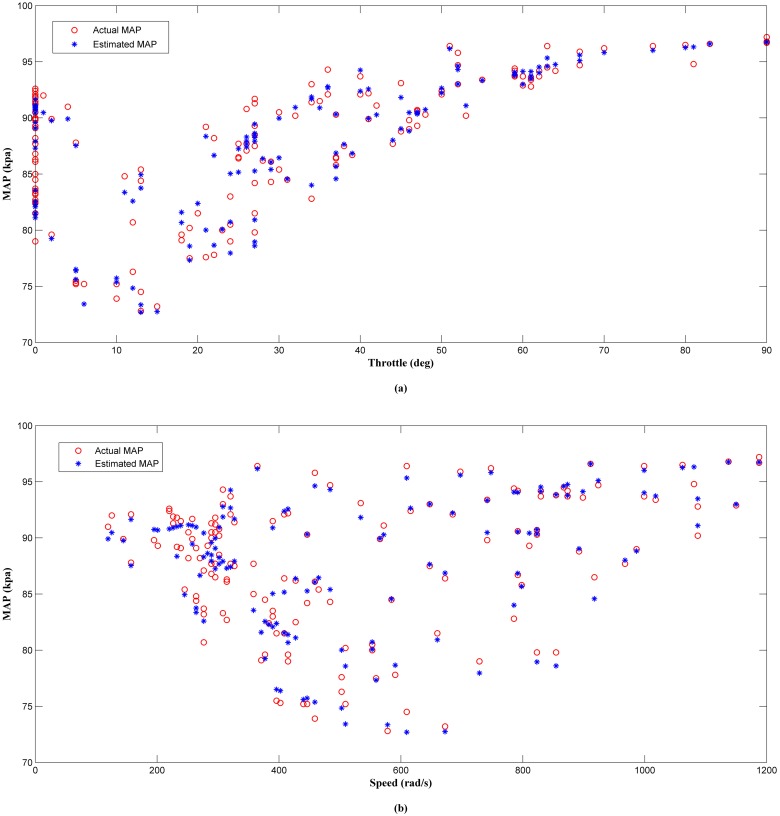
Comparison of the MAP estimator output (LM+BR+PSO_a_) and actual MAP as function of (a) throttle and (b) speed.

**Fig 12:**
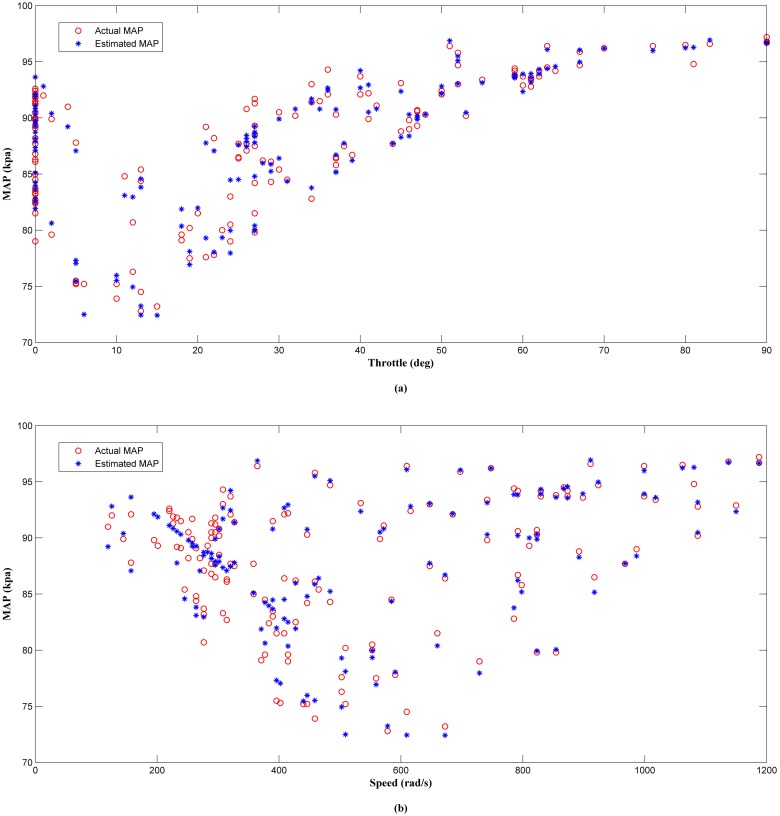
Comparison of the MAP estimator output (LM+BR+PSO_b_) and actual MAP as function of (a) throttle and (b) speed.

**Fig 13:**
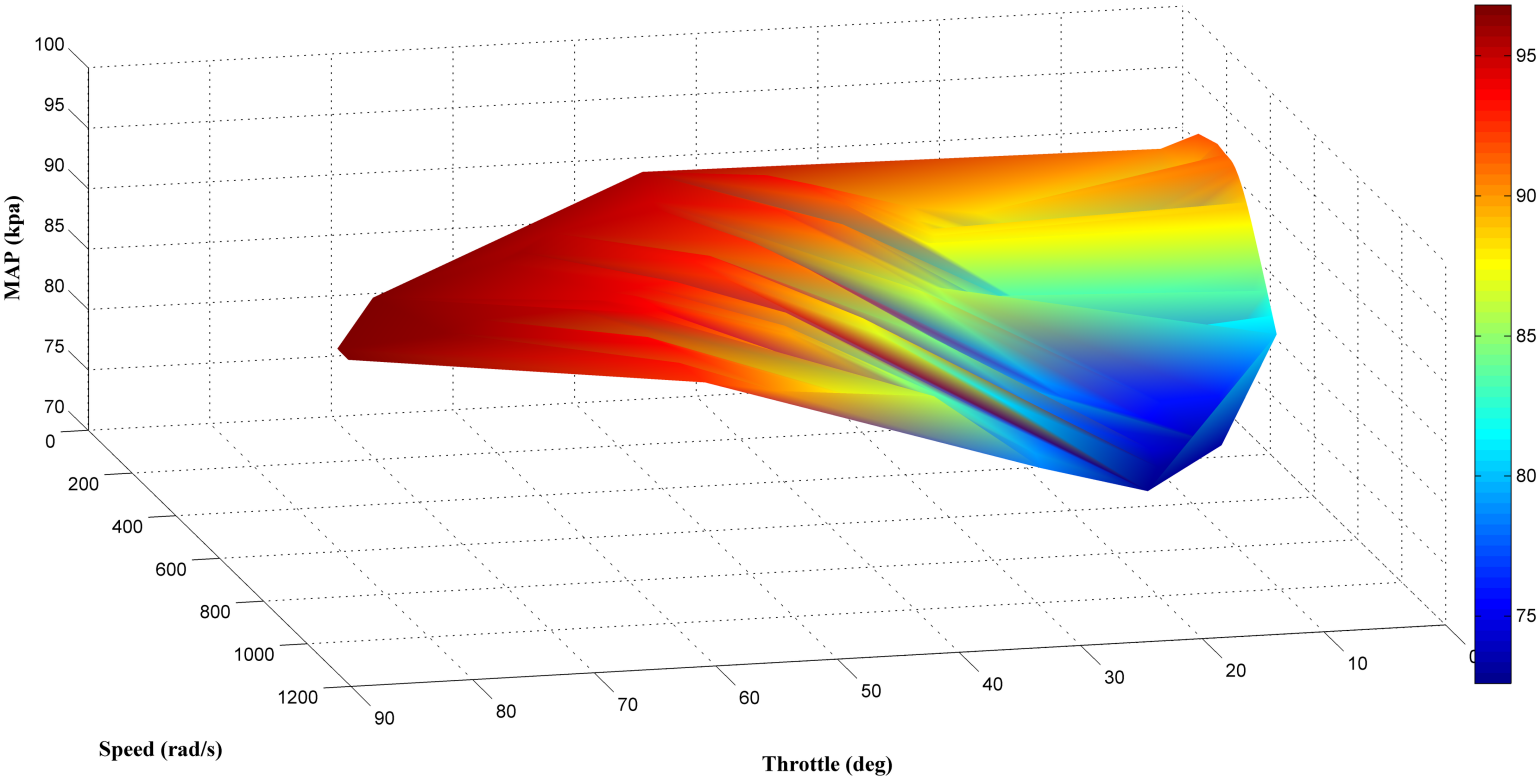
3D Surface plot of the trained estimator (LM+BR+PSO_b_).

The results of the offline analysis are summarized in Table 4. It can be seen that the estimator (LM+BR+PSO_b_) produced the best prediction of the MAP with the smallest *MSE* value of 1.9863 compared to the other four algorithms. This was followed by LM+BR+PSO_a_ (2.2435), LM+BR (3.3293), LM (4.2509) and PSO which has the worst performance (11.3352). The outcome of this analysis shared a same trait as in simulation work. This results were also supported by the value of *R*^*2*^ between the test data and the predicted outputs which follow the same trend as the *MSE*. This can be observed in the scatter plot as shown in Figs 8, 9, 10, 11 and 12. In addition, a 3D plot can also be seen in Fig 13 which illustrate the relation between the inputs and the estimated output of the best trained estimator (LM+BR+PSO_b_).

**Table 4:** Performance of the MAP estimator for the RFIS in 20 runs.

*Training Algorithm*	*Average test MSE*	*Average R^2^*	*Average computation time(s)*
*LM*	*4.2509*	*0.8839*	*101.3251*
*LM+BR*	*3.3293*	*0.9091*	*305.1631*
*PSO*	*11.3352*	*0.7104*	*190.1241*
*LM+BR+PSO_a_*	*2.2435*	*0.9387*	*381.0012*
*LM+BR+PSO_b_*	*1.9863*	*0.9458*	*509.2051*

**Fig 14:**
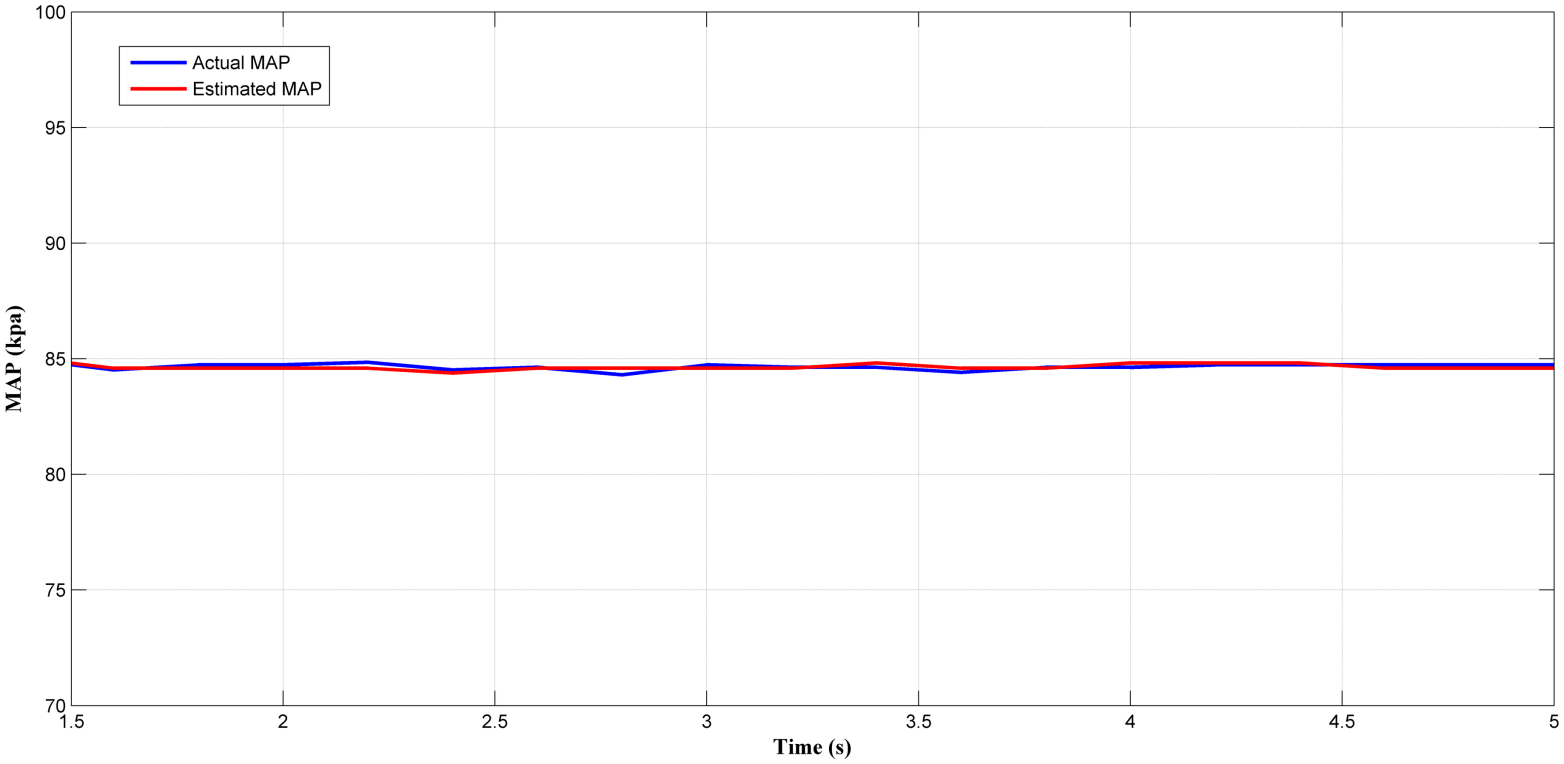
Comparison between estimated and measured MAP at 367 rad/s and 20° of throttle angle.

**Fig 15:**
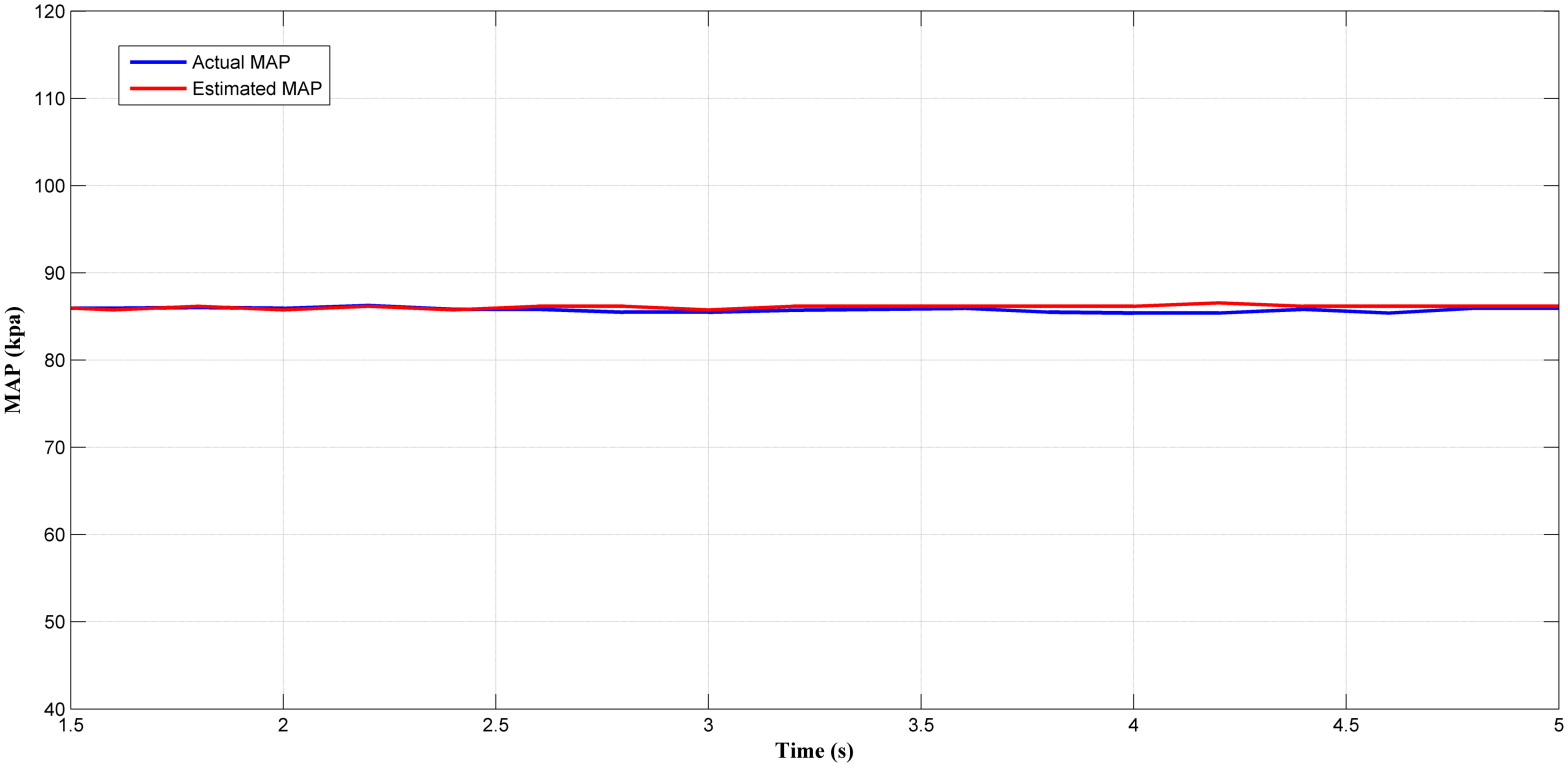
Comparison between estimated and measured MAP at 785 rad/s and 40° of throttle angle.

**Fig 16:**
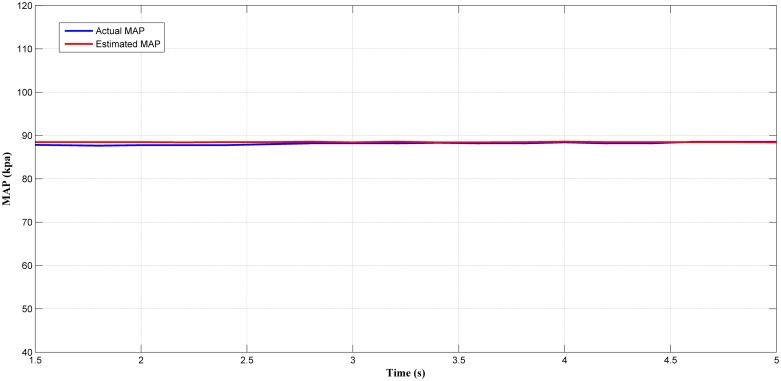
Comparison between estimated and measured MAP at 942 rad/s and 60° of throttle angle.

**Fig 17:**
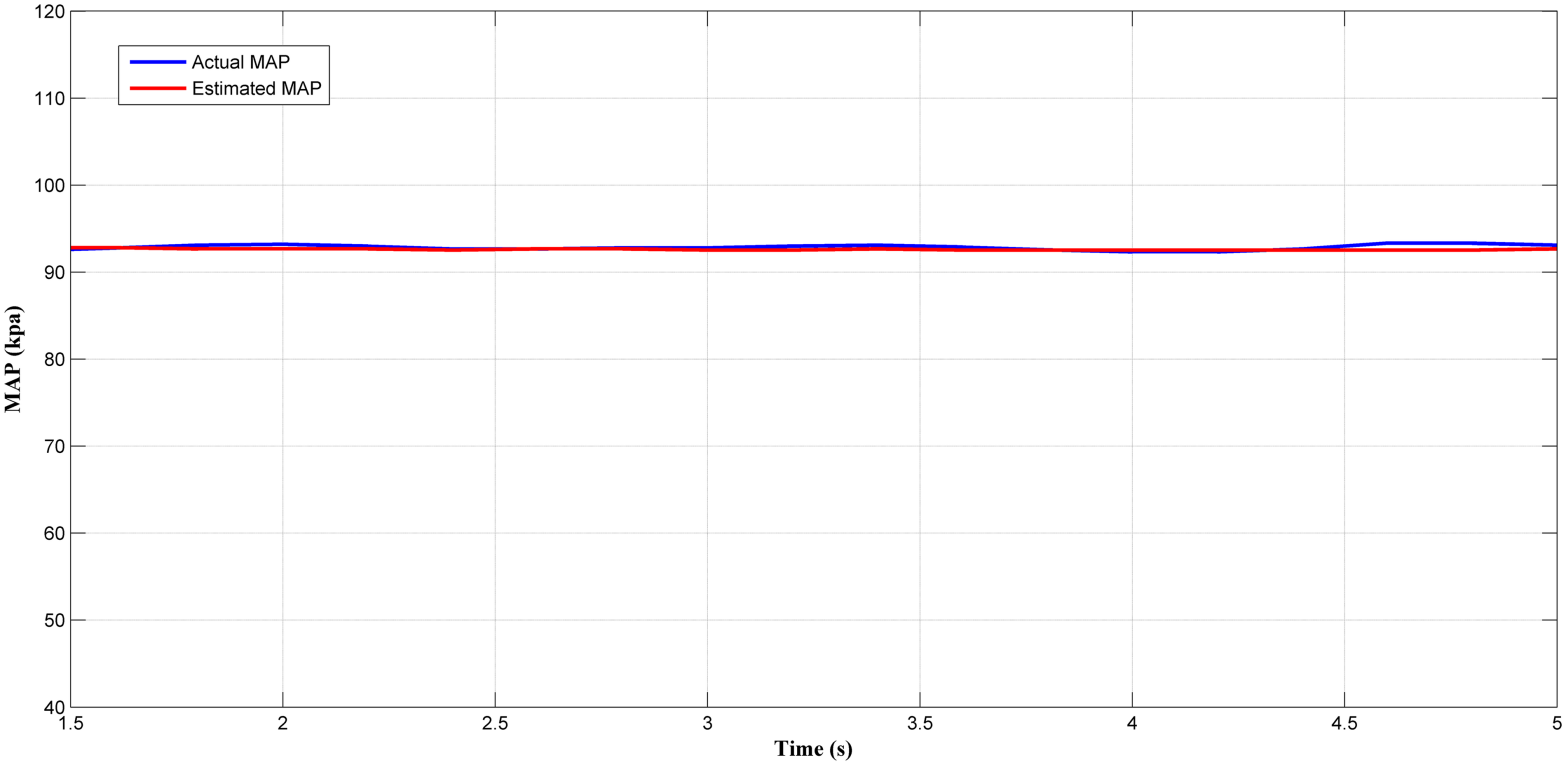
Comparison between estimated and measured MAP at 1100 rad/s and 80° of throttle angle.

Next, the selected best estimator (LM+BR+PSO_b_) output was tested in a steady-state condition. Figs 14, 15, 16 and 17 showed the plotted of the estimated MAP against the actual MAP in steady-state condition. The test results are summarized in Table 5. According to Table 5, the *MSE* decreases as the throttle and engine speed increased. This indicate that the predicted output at lower operating range was less accurate if compared to that higher operating range. This can be observed in Figs 14–17. However, the different of *MSE* value between each test was small. This showed the capability of the MAP estimator when the RFIS was operated in steady-state condition.

**Table 5:** MSE of the MAP estimation at different engine speed and throttle angle.

*Engine Speed (rad/s)*	*Throttle angle (degrees)*	*MSE*
*367*	*20*	*1.4768*
*785*	*40*	*1.3086*
*942*	*60*	*1.2709*
*1100*	*70*	*1.2366*

**Fig 18:**
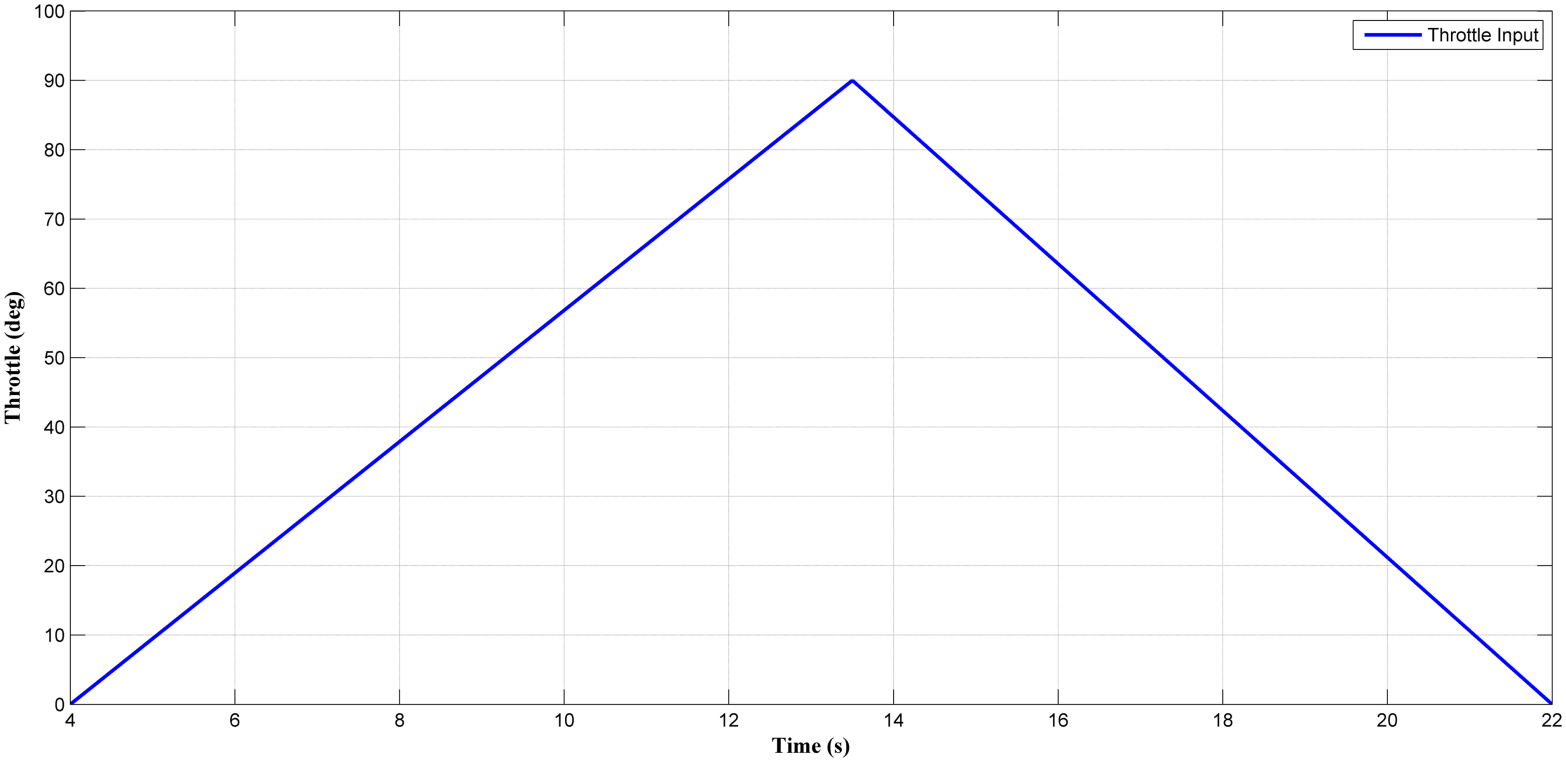
Throttle transient input in the RFIS.

**Fig 19:**
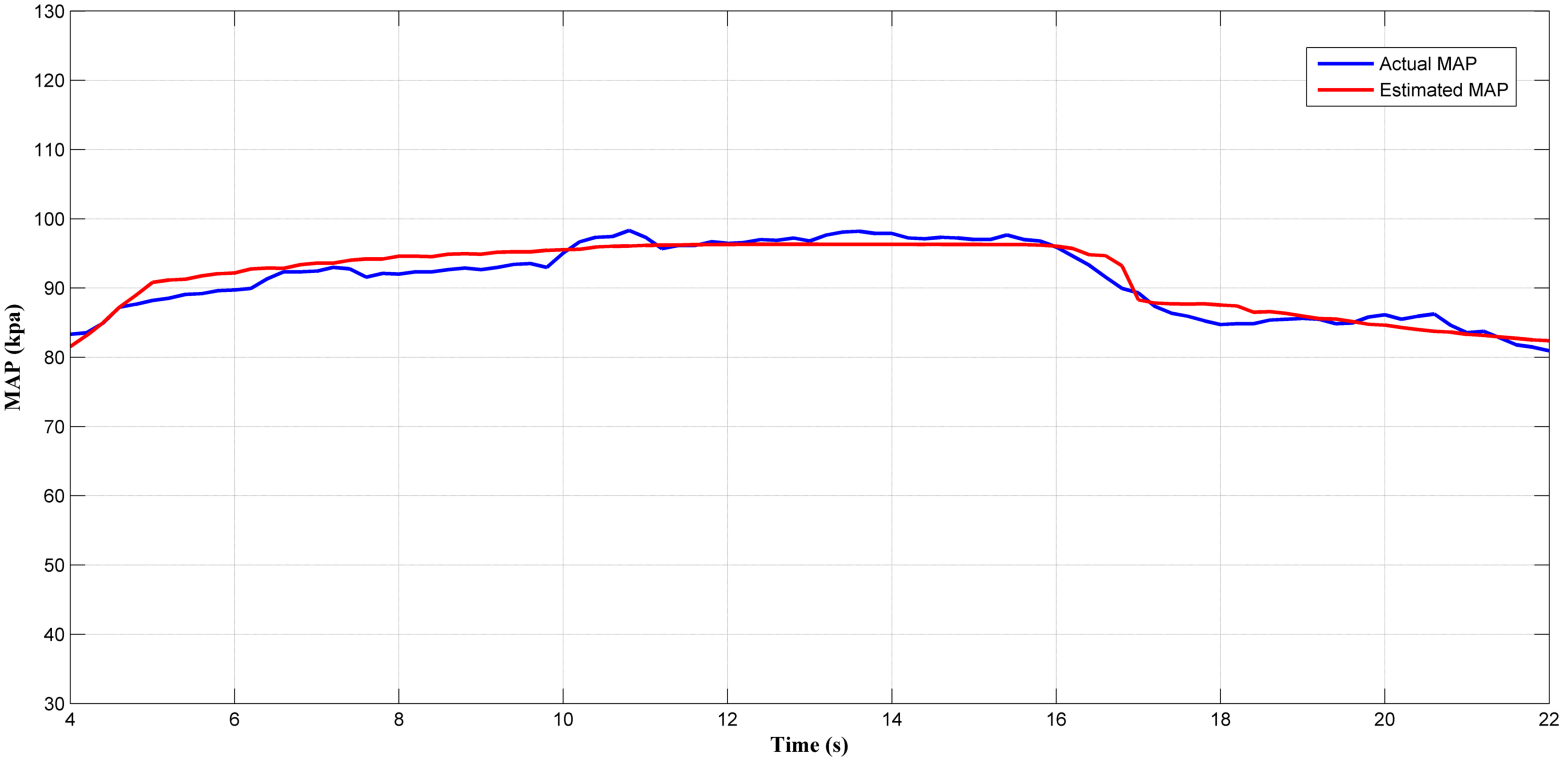
Comparison between estimated and measured MAP in transient operation.

Next, the selected estimator (LM+BR+PSO_b_) was tested in a transient condition. A fast transient conditions was induced by throttle operation as shown in Fig 18. The estimated output and the actual output are shown in Fig 19 with *MSE* of 4.534. From observation in Fig 19, the predicted MAP value follows the trend of the actual MAP. From this, the MAP estimator can certainly react to throttle transient but with less accuracy especially when the throttle increase (15° to 80°) and decrease from wide open throttle (90°) to lower throttle (15°). Thus, the efficiency of the estimator in transient condition in the RFIS was not as good as the one in the steady-state condition. Nevertheless, this result proved that the MAP estimator can also be used in the transient engine operation with a drop in small accuracy.

## Conclusions

The proposed estimator presented in this paper managed to estimate the absolute pressure of the intake manifold close to the actual value in both simulation and experimental work. The first and the second variant of the hybrid algorithm which consists of LM, BR and PSO showed a significant improvement compared to the standard PSO and LM algorithm regardless of the computation time. Based on the results in 20 runs, the second variant of hybrid algorithm (LM+BR+PSO_b_) yields a better network performance with a mean squared error (*MSE*) of 0.0871 by estimating the MAP closely to the simulated MAP values compared to using the first variant of hybrid algorithm (*MSE* of 0.1088), LM (*MSE* of 6.1446), LM with BR (*MSE* of 1.7704) and PSO (*MSE* of 249.4320) alone.

By using a valid experimental training data, the estimator network that trained with the second variant of the hybrid algorithm (LM+BR+PSO_b_) showed the best performance, with *MSE* of 1.9863, among other algorithms when used in an actual retrofit fuel injection system (RFIS). The performance of the estimator was also validated in steady-state and transient condition by showing a closer MAP estimation to the actual value. Nevertheless, the performance of the estimator will likely decrease due to aging effect of the engine which cause a degradation in its performance. Hence, the estimator need to be retrained again.

## Supporting information

S1 Table Data collection for retrofit fuel injection system (RFIS). (XLSX)
